# Commentary: Diagnostic utility of hematological and biochemical markers for cystic echinococcosis in Tibetan patients of Sichuan, China

**DOI:** 10.3389/fcimb.2025.1700616

**Published:** 2025-11-11

**Authors:** Qing-Bao Jiang, Guo-Ming Zhang

**Affiliations:** 1 Department of Laboratory Medicine, Daqing Hospital of Traditional Chinese Medicine, Daqing, Heilongjiang, China; 2 Department of Laboratory Medicine, Shuyang Hospital, The Affiliated Shuyang Hospital of Xuzhou Medical University, Shuyang, Jiangsu, China

**Keywords:** cystic echinococcosis, hematological marker, biochemical markers, laboratory diagnosis, plasminogen time

## Introduction

Cystic echinococcosis (CE) remains a major neglected zoonosis and a persistent diagnostic and therapeutic challenge, particularly in endemic and resource-limited settings. Diagnosis is often delayed because of its prolonged asymptomatic phase and the nonspecific nature of early clinical and laboratory findings. Although ultrasound is considered the first-line imaging modality, differentiating active from inactive cysts and evaluating treatment response remain difficult. Moreover, serological assays vary in sensitivity and specificity across cyst stages, leading to inconsistent results. Therapeutic management is also complex and requires an individualized approach combining surgery, percutaneous drainage, or benzimidazole therapy. These challenges highlight the need for accessible laboratory indicators to complement imaging and guide clinical decision-making in endemic areas (Shafiei et al., 2024).

## General comments

We read with great interest the article by Ma et al. that evaluated routine hematological and biochemical parameters as diagnostic tools for CE in Tibetan patients in Sichuan (Ma et al., 2025). The authors are to be commended for addressing a neglected disease in a resource-constrained population and for seeking inexpensive biomarkers that could complement imaging and serology. Nevertheless, several methodological and statistical concerns limit the strength of their conclusions and should be carefully considered before these findings are applied to clinical or public health practice.

### First, concerns with the control group

The investigators included 83 confirmed cases of CE and 45 healthy controls matched for age and sex to minimize demographic confounding factors (Ma et al., 2025). Although such exclusions enhance internal validity, they substantially reduce external validity because they fail to reflect the diagnostic context of endemic regions, where eosinophilia and coagulation abnormalities frequently occur in other helminthic or hepatic disorders. Eosinophilia is a well-recognized but nonspecific response to a wide range of parasitic infections, allergic diseases, and immune-mediated conditions (Huang and Appleton, 2016). Similarly, several helminthic hepatopathies, such as schistosomiasis and fascioliasis, can alter coagulation parameters [e.g., prolonged prothrombin time (PT) or activated partial thromboplastin time (APTT)] through hepatic injury or inflammatory consumption (Ma et al., 2025). Consequently, by excluding diseased controls, the study design likely overestimates the discriminative ability of the PT and eosinophil percentage (EOS%) to differentiate CE from other endemic infections or liver diseases.

### Second, diagnostic circularity and hepatic dysfunction

Over 90% of patients with CE had liver involvement on imaging, and prolonged PT was interpreted as a hallmark predictor. However, PT prolongation is likely a downstream marker of hepatic dysfunction rather than a disease-specific indicator of CE. This introduces a form of circular reasoning: the case definition included imaging evidence of hepatic cysts, and the predictive marker (PT) reflects impaired hepatic function. As Nunnari et al. emphasized, PT abnormalities are typical in advanced hepatic echinococcosis but cannot distinguish CE from other causes of liver impairment (Nunnari et al., 2012).

### Third, there is an implausible confidence interval and a lack of AUC comparisons

The most striking issue concerns the receiver operating characteristic (ROC) results. PT alone achieved an area under the curve (AUC) of 0.969 [95% confidence interval (CI): 0.940–0.997]. The combination of PT and EOS% yielded an AUC of 0.982, with a reported 95% CI of 0.902–1.001 (Table 5, Figure 2) (Ma et al., 2025). An upper bound above 1.0 is mathematically impossible and indicates either a computational or reporting error. Furthermore, the 95% CIs for PT and PT + EOS% overlap substantially. Overlapping intervals indicate that no statistically significant difference can be inferred. The appropriate method for testing the difference between correlated ROC curves is the DeLong nonparametric test or, alternatively, bootstrap resampling. This test yields a *Z* statistic and *p*-value for the AUC difference. Without such a test, claims that PT + EOS% outperforms PT alone are unsupported. We strongly recommend recalculating all the AUCs with corrected CIs, ensuring that they fall within [0, 1], and formally reporting the AUC with its standard error, *Z*-value, and *p*-value (DeLong et al., 1988).

### Fourth, descriptive statistics and distributional assumptions

Table 2 presents laboratory values as the means ± standard deviations (SDs) (Ma et al., 2025). However, several variables exhibit extreme skewness, as evidenced by large SDs relative to the means (e.g., total bilirubin 32.2 ± 68.3 μmol/L; gamma-glutamyl transferase 125 ± 178 U/L; alkaline phosphatase 204 ± 278 U/L). These distributions are clearly non-normal. Reporting means in this context is misleading, as it obscures the central tendency and exaggerates variability. For skewed data, the standard approach is to present medians with interquartile ranges (IQRs). Nonparametric tests (Mann–Whitney *U*) should be applied instead of *t*-tests unless appropriate transformations (e.g., log) are performed. Moreover, providing effect sizes with 95% CIs (e.g., median difference or Hodges–Lehmann estimator) would convey more clinically meaningful contrasts between groups (Altman and Bland, 1996). Adopting these practices would align the study with STARD guidelines for reporting diagnostic accuracy (Bossuyt et al., 2015).

### Fifth, regression modeling and risk of overfitting

In multivariate logistic regression, PT emerged as the sole independent predictor, with an odds ratio exceeding 50 (95% CI: 6.18–429.34). This extreme effect size with a wide CI suggests the instability of the model, likely due to the small sample size and collinearity among hepatic markers. Internal validation (bootstrap resampling or cross-validation) and calibration metrics (e.g., Hosmer–Lemeshow test and calibration plots) were not reported. Without these factors, the predictive model risks overfitting and may not be generalizable beyond the study cohort (Steyerberg et al., 2001).

### Sixth, the clinical implications of sensitivity and specificity

At the chosen cutoff (>12.2 s), PT achieved a sensitivity of 85.5% and a specificity of 97.5%. EOS% had a sensitivity of only 55.4%, limiting its value as a screening tool. In endemic populations, sensitivity is often prioritized to avoid missed cases, even at the expense of specificity (Tamarozzi et al., 2021). The combined model, despite its slightly higher AUC, does not clearly address this trade-off. A decision-analytic approach (e.g., net reclassification improvement or decision curve analysis) would help determine whether EOS% meaningfully adds clinical value (Vickers and Elkin, 2006).

### Seventh, generalizability and feasibility

The authors acknowledge that their hospital-based design limits generalizability. Indeed, hematological norms vary with altitude, ethnicity, and nutritional status and are not fully controlled here. Moreover, routine PT testing requires coagulation analyzers, reagents, and trained staff, which may not be consistently available in remote Tibetan settlements. In contrast, ultrasound screening has been endorsed by the World Health Organization (WHO) as the most practical community-level tool for CE surveillance (Rinaldi et al., 2014). Therefore, while PT and EOS% may serve as adjunctive markers, their feasibility as frontline screening tools remains uncertain.

## Discussion

Ma et al. highlighted the potential role of routine laboratory markers in CE diagnosis. However, critical issues must be addressed (1): correction of the impossible 95% CI exceeding 1.0 (2); formal statistical testing of AUC differences via the DeLong or bootstrap methods; and (3) appropriate use of medians and nonparametric tests for skewed data. Without these corrections, the claim that PT + EOS% substantially improves diagnostic accuracy over PT alone is not justified. We commend the authors for their contribution to an under-researched field but recommend that future studies incorporate diseased controls, adopt robust statistical methodology, and validate findings in community-based cohorts. Only then can PT and EOS% be considered reliable, generalizable biomarkers for CE in endemic regions.
